# What Can Computational Models Contribute to Neuroimaging Data Analytics?

**DOI:** 10.3389/fnsys.2018.00068

**Published:** 2019-01-10

**Authors:** Oleksandr V. Popovych, Thanos Manos, Felix Hoffstaedter, Simon B. Eickhoff

**Affiliations:** ^1^Institute of Neuroscience and Medicine - Brain & Behaviour (INM-7), Research Centre Jülich, Jülich, Germany; ^2^Institute of Systems Neuroscience, Medical Faculty, Heinrich-Heine University Düsseldorf, Düsseldorf, Germany

**Keywords:** neuroimaging, resting state, mathematical models, brain dynamics, functional connectivity, simulation, high-performance computing

## Abstract

Over the past years, nonlinear dynamical models have significantly contributed to the general understanding of brain activity as well as brain disorders. Appropriately validated and optimized mathematical models can be used to mechanistically explain properties of brain structure and neuronal dynamics observed from neuroimaging data. A thorough exploration of the model parameter space and hypothesis testing with the methods of nonlinear dynamical systems and statistical physics can assist in classification and prediction of brain states. On the one hand, such a detailed investigation and systematic parameter variation are hardly feasible in experiments and data analysis. On the other hand, the model-based approach can establish a link between empirically discovered phenomena and more abstract concepts of attractors, multistability, bifurcations, synchronization, noise-induced dynamics, etc. Such a mathematical description allows to compare and differentiate brain structure and dynamics in health and disease, such that model parameters and dynamical regimes may serve as additional biomarkers of brain states and behavioral modes. In this perspective paper we first provide very brief overview of the recent progress and some open problems in neuroimaging data analytics with emphasis on the resting state brain activity. We then focus on a few recent contributions of mathematical modeling to our understanding of the brain dynamics and model-based approaches in medicine. Finally, we discuss the question stated in the title. We conclude that incorporating computational models in neuroimaging data analytics as well as in translational medicine could significantly contribute to the progress in these fields.

## 1. Data-Driven Approach

Advances in neuroimaging techniques and data analytics methods have led to a rapid advancement in the description of structural and functional properties of brain networks. The anatomical architecture of brain networks, i.e., their structural connectivity (SC), can be inferred from diffusion weighted magnetic resonance imaging (dwMRI). In particular, fiber tracts reconstructed through tractography algorithms can serve as proxies for physical connections between brain regions at the meso- and macroscopic level (though see Jones et al., 2013; Thomas et al., 2014; Maier-Hein et al., 2017 for a discussion on the limitations of fiber reconstruction from dwMRI). Such structural information thus contributes a description of the underlying physical organization of the brain on top of which the time dynamics of neuronal activity can emerge. The latter can be measured by indirect, blood flow-based methods such as functional MRI (fMRI) and positron emission tomography (PET) as well as more direct recording of neuronal activity through electroencephalography (EEG), magnetoencephalography (MEG) or as local field potential (LFP) measured by implanted electrodes. While these methods differ substantially with respect to their spatial and temporal resolution (cf. Horwitz et al., 2000; Bandettini, 2009), they can all be used in two fundamental contexts in task-related and task-free examinations. Classically, subjects perform an active sensory, cognitive or motor paradigm while brain activity is recorded. This allows to probe context-dependent modulations of regional activity and network interactions, reflecting the effects of the respective tasks on functional and effective connectivity (Bressler and Menon, 2010; Park and Friston, 2013).

While massively growing our knowledge on functional specialization and integration over more than two decades, task-based imaging also suffers from several drawbacks. Brain activity in a task setting is bound to the particular experimental context and hence limited in generalizability, re-usability as well as in practicality for imaging large cohorts or a clinical setting. Consequently, much focus has been devoted more recently to the acquisition and analysis of what has been termed the “task-free” or “resting-state” activity of the brain. Making use of the constantly ongoing mental processes and the fact that brain networks hence coordinate their activity also in the absence of an explicit behavioral task, resting-state imaging (Biswal et al., 1995) has yielded new perspectives in the study of large cohorts and patient populations as well as establishing data-sharing in the field of neuroimaging. Consequently, large databases collected thousands of brain imaging records and behavioral information (Van Essen et al., 2013; Caspers et al., 2014). In the most common approach, i.e., resting-state fMRI, the temporal fluctuations of the blood oxygen level-dependent (BOLD) signals are related to each other by clear and robust correlation (e.g., by using Pearson correlation coefficient) across what are assumed to be functionally related brain regions coordinating their activity during sensory, motor or cognitive brain functions, i.e., functional connectivity (FC) between them (Biswal et al., 1995; Park and Friston, 2013; Varikuti et al., 2017).

Resting-state FC analysis has without doubt revolutionized the field of human brain imaging and has led to the presence of many new insights into inter-individual variability and the pathophysiology of brain disorders. For example, differences in the group-average resting-state FC patterns have been shown for disorders such as Parkinson's and Alzheimer's disease, stroke, depression and schizophrenia (Sorg et al., 2007; Konrad and Eickhoff, 2010; Tahmasian et al., 2015). Moreover, it has been demonstrated, that individual patterns of BOLD correlations in the absence of a structured task allow for the individual classification of previously unseen subjects as patients or healthy controls with good accuracy (Arbabshirani et al., 2017; Pläschke et al., 2017). Together with the fact that resting-state measurements are attainable even in comparably ill patients, this opens an avenue for a clinical application toward objective diagnosis and may even allow to provide prognoses of disease development on an individual basis (Woo et al., 2017; Yahata et al., 2017). Finally, investigating FC patterns of spontaneous brain activity at the single-subject level, individualized targets for interventional approaches using invasive or non-invasive neuromodulations may provide a road to more individualized, neurobiologically informed treatments (Fox et al., 2014).

In healthy subjects, resting-state FC can be used to build data-driven connectome–based predictive models to differentiate between subjects and to predict individual behavior from the brain activity (Shen et al., 2017). Robust and reliable individual variability of the FC profiles at “rest” permits a relatively accurate identification of individual subjects from a large group (Finn et al., 2015). The contemporary research in this framework is focused on prediction of fluid intelligence of individual subjects, performance in specific tasks, personality traits and brain aging from resting-state fMRI data (Dosenbach et al., 2010; Finn et al., 2015; Dubois and Adolphs, 2016; Tavor et al., 2016; Liem et al., 2017). This also has an important clinical relevance, for instance, in distinguishing between normal aging and disease.

In spite of its undisputed success, however, resting-state fMRI is also a field marked by substantial controversy over the appropriate analyses methods and the interpretation of observed patterns. For the task-based imaging there could exist a relatively clear time frame and related expectations, where and what is going to happen in the brain and its relation to behavioral states across subjects. For the resting-state measurements, the absence of an external reference by experimentally defined time-points and behavioral states together with enhanced inter-individual variability renders the whole field devoid of any gold-standard or ground-truth. This makes the resting-state data processing and analysis and interpretation of results even more challenging than for the task-based paradigm.

It may hence not surprise that many of the most cited neuroimaging papers over the last years pertain to the characterization of (potential) artifacts, evaluation of processing or analysis methods and inferences that can be drawn from these (Murphy et al., 2013; Satterthwaite et al., 2013; Power et al., 2014; Salimi-Khorshidi et al., 2014). For example, it is generally agreed that artificial sources of variance caused by subject movement as well as by various sources of physiological noise (such as heartbeat, breathing) may strongly influence the observed BOLD correlations and need to be accounted for. How this goal is best achieved, though, is a matter of conjecture as there is no gold standard given that we don't know how the BOLD signal would look like without these influences. Consequently, methods for characterizing and removing what should be considered structured noise within resting-state time series have, e.g., proxied the effects of movement for example by differences between high- and low-moving subjects (Satterthwaite et al., 2013). There is an ongoing discussion on how to deal with global signal changes, as it can be shown that when these are not accounted for, virtually all brain regions exhibit functional correlations with each other while removal of global signals leads to pronounced anti-correlations (Varikuti et al., 2017). The latter are questioned as spurious by some (Murphy et al., 2009) and highlighted as antagonistic large-scale systems by others (Fox et al., 2009). Finally, in particular in the context of predicting individual phenotypes and ultimately precision medicine, one of the key prerequisites is to reduce the dimensionality from all possible interactions among >200.000 voxels onto a tractable number of region-by-region connections. This is usually done through referencing to a brain atlas providing the respective parcellation, but unfortunately, there is no general consensus as to which parcellation would be most appropriate, see the papers (Thirion et al., 2014; Eickhoff et al., 2018a,b) for review and references therein. In fact, both the approach for atlas generation with respect to imaging modalities and parcellation techniques (resting-state, structure, geometry, independent/principal components analysis (ICA/PCA), clustering etc.) and the ensuing granularity vary considerably across studies performing connectome-based resting-state analyses.

In summary, while the potential of resting-state fMRI to contribute to our understanding of the normal and diseased brain is extremely high, many questions on how to attain this goal are still unresolved.

## 2. Model-Based Approach

The achievements of the data-driven approach discussed above can significantly be extended if mathematical models get involved in the analysis to gain possible mechanistic explanations of the dynamics and properties of the brain resting-state networks (RSN). A thorough exploration of possible dynamical repertoires that the models can support and their relation to the phenomena observed in the data can help to formulate predictions and hypotheses that can serve as a basis for further experimental investigation and data analysis. A number of models of different complexity have been suggested for RSN neural population dynamics including phase oscillators (Cabral et al., 2011; Ponce-Alvarez et al., 2015), limit-cycle oscillators (Bettinardi et al., 2017; Deco et al., 2017), mean-field and spiking neuron models taking into account contributions of excitatory and inhibitory neurons located within the network nodes (Deco and Jirsa, 2012; Deco et al., 2014b; Glomb et al., 2017), and others (Deco et al., 2008; Sanz-Leon et al., 2015). The individual nodes can be coupled to brain networks, where the inter-node connections can be derived from empirical data, which was coined as the brain structural connectome (Sporns et al., 2005). Such SC obtained from diffusion weighted imaging and tract tracing studies can be used to build a graph of the brain network defining an underlying topological structure of the model network (Knock et al., 2009; Bullmore and Bassett, 2011; Park and Friston, 2013).

The model parameters can be calibrated in such a way that the model dynamics closely replicates that of the brain networks extracted from the empirical data. Realistic power spectra and fluctuations of BOLD signal were obtained from the models simulating underlying electrical activity of the neuronal populations (Ghosh et al., 2008; Deco et al., 2009; Deco and Jirsa, 2012; Schirner et al., 2018), where an advance modeling can also be used to infer the neurophysiological mechanisms underlying neuroimaging signals (Schirner et al., 2018). At this, parameters of noise intensity, time delay in coupling, global coupling strength, and excitation-inhibition balance may play a crucial role. It was found that there can exist optimal parameter settings, where the matrix of empirical FC strongly correlates with that calculated from the data generated by the model (Deco and Jirsa, 2012; Deco et al., 2013; Nakagawa et al., 2013). The maximal correspondence between model and empirical data can emerge when the model is close to a critical point at which the system may undergo a bifurcation, a sudden qualitative change of its dynamics and engage in a multistable regime that is highly sensitive to perturbations (Deco et al., 2013; Golos et al., 2015; Cocchi et al., 2017). Noise-driven exploration of the state space in the vicinity of attractors allows the emergence of dynamics observed in RSN activity (Ghosh et al., 2008; Deco et al., 2012, 2013; Golos et al., 2015).

FC patterns may strongly vary in time as we discuss below, and the static description of FC matrix obtained over long epochs (≥5 min) is distinguished by so-called dynamic FC reflecting time variations of correlations and characterized by the ongoing switching of network patterns (Hutchison et al., 2013; Allen et al., 2014; Cabral et al., 2017b). Analysis of dynamic FC for patients with schizophrenia and bipolar disorder has revealed different switching regimes as compared to healthy controls (Damaraju et al., 2014; Rashid et al., 2014), which cannot be obtained by the static connectivity analysis. The extraction, analysis, interpretation and application of dynamic FC are in the focus of investigation nowadays, see the recent comprehensive review (Preti et al., 2017).

In the framework of model-based approach, several recent studies (Hansen et al., 2015; Deco and Kringelbach, 2016; Deco et al., 2017; Jobst et al., 2017) modeled dynamic FC and showed that non-stationary connectivity dynamics can demonstrate a rich spatio-temporal structure and fast switching between a few discrete states as observed in empirical data. These studies also reported that the brain at rest operates at a maximal level of metastability (Deco and Kringelbach, 2016; Deco et al., 2017; Jobst et al., 2017), which can be referred to as an out-of-equilibrium state characterized by a pronounced variability of phase configurations of the system and switching between different regimes of collective dynamics. It was suggested to be measured by the standard deviation of the Kuramoto order parameter reflecting the extent of synchronization in the networks across time (Deco and Kringelbach, 2016; Deco et al., 2017; Jobst et al., 2017). The concept of maximal metastability for the RSNs in the first place implies a state of maximum network switching and enhanced variability of synchronization in resting-state brain activity. Metastability is a fundamental property of transient brain dynamics and cognitive processes such as sequential decision making which can be modeled by a stable heteroclinic cycle involving complex metastable brain states (Rabinovich et al., 2008; Tognoli and Kelso, 2014). Therefore, fitting the extent of metastability and the properties of dynamic FC of the model to empirical data can provide an additional tool for validation and optimization of whole-brain models (Deco and Kringelbach, 2016; Deco et al., 2017; Jobst et al., 2017).

The model-based approach can effectively be used for addressing neuropsychiatric disorders aiming to describe disease mechanisms (Deco and Kringelbach, 2014; Stephan et al., 2015). One example is schizophrenia which can be viewed as a brain network disorder (Friston and Frith, 1995; Calhoun et al., 2009; Lynall et al., 2010; van den Heuvel and Fornito, 2014). Simulations of whole-brain computational models demonstrated that clinical symptoms and changes in FC patterns observed in empirical data can be accounted for by a variation (decrease) of the global coupling strength in the model (Cabral et al., 2012b, 2013). On the other hand, the average power and variance of the global brain signal may increase in schizophrenia, which was replicated by dynamics of a computational model when local or global coupling strength increases (Yang et al., 2014; Anticevic et al., 2015). Modeling approaches can also be used to illustrate how the changes in SC due to long-term deep brain stimulation (DBS) in patients with Parkinson's disease (PD) can be reflected in resting-state brain dynamics and FC (van Hartevelt et al., 2014). Whole-brain computational models can help to reveal acute effects of DBS on resting-state brain dynamics when comparing DBS-On and DBS-Off regimes (Saenger et al., 2017). When comparing model parameters of local and global coupling as well as conduction velocity fitted to the functional data of stroke patients and healthy controls, a significant difference between these two parameter sets can be observed (Falcon et al., 2016). Furthermore, the coupling parameters correlated with long-term motor gains during recovery after stroke, which indicates that the model parameters can serve as biomarkers with potential predictive power for recovery after stroke (Falcon et al., 2016).

The potential of brain network modeling for diagnose, prognosis and treatment of neurological and psychiatric diseases is probably the most important perspective of this approach. The growing field of precision medicine is directed toward disease prevention and more predictive and personalized medicine as opposed to the treatment of patients mostly based on their segregation with respect to disease type or subtype (Duffy, 2016). Such personalized approach to the medical treatment would in particular require an involvement of personalized predictive computational models of individual patients. In this framework the concept of the virtual epileptic patient has recently been proposed (Jirsa et al., 2017; Proix et al., 2017), where the development of a personalized mathematical model for an epileptic patient was illustrated step by step. Such a model is based on the personalized SC and other indicators (e.g., lesions, epileptogenic zones, etc) and can be validated and optimized to reflect, explain and predict epileptic seizure propagation (Proix et al., 2017). Systematic explorations of the model parameter space can give an insight into how the clinically relevant parameters, such as neuronal excitability and coupling strength, might influence the number of seizures, their localization and propagation. Although the relation between the changes of the model parameters and impacts of the clinical intervention is not always evident, the models can nevertheless serve as a test bed for clinical hypothesis testing and thus provide the clinicians with an additional tool for hypothesis building and decision-making at brain intervention strategies. The knowledge obtained from the model-based investigation and data fitting, for example, for localization and extent of the epileptogenic zone can be combined with a data-driven approach and the experience of clinicians. This can contribute to the improvement of diagnosis and surgery outcome as well as to development of novel therapies (Jirsa et al., 2017; Proix et al., 2017).

Applying the model-based approach, brain stimulation techniques have been developed for counteracting abnormal neuronal synchronization characteristic for some neurological disorders including PD and tinnitus by desynchronization (Tass, 2003; Tass and Majtanik, 2006; Popovych and Tass, 2012; Ebert et al., 2014; Manos et al., 2018a,b). Desynchronizing stimulation techniques have been designed, tested and optimized with the help of mathematical models of neuronal populations. Parameters of stimulation intensity, timing and shape of the stimulation signal have systematically been varied and evaluated, which is hardly feasible in experimental and clinical setting. The hypotheses and predictions drawn from the modeling studies have successfully been tested in pre-clinical and clinical studies (Tass et al., 2012a,b; Adamchic et al., 2014) for different stimulation modalities including electrical DBS for PD and noninvasive acoustic stimulation for tinnitus.

A recent shift toward a closed-loop stimulation paradigm for DBS (Little et al., 2013; Rosa et al., 2015) has led to a more efficient counteracting of disease symptoms. The same clinical effects can be achieved for a significant reduction of the amount of the delivered stimulation current if the stimulation is administered when necessary in a demand-controlled way. This was preceded by theoretical development and modeling of closed-loop stimulation methods (Rosenblum and Pikovsky, 2004; Popovych et al., 2005, 2006). Model-based approach can further be used to optimize and adapt the stimulation techniques for clinical application, where they can be tested for realistic DBS signals (Popovych et al., 2017a,b; Popovych and Tass, 2018). Optimal parameter values can be found based on a thorough scan of parameter space of the models.

As for the research driven by the data, the model-based approach is not limited to the investigations of diseased states. Large-scale whole-brain computational models can provide an insight into how resting-state brain activity and FC patterns may evolve in aging (Nakagawa et al., 2013; Naik et al., 2017). Dynamics of the model and its parameters, for example, the coupling strength and the delay in coupling demonstrate consistent differences when comparing groups of young and old adults with high and low performance in cognitive tasks (Naik et al., 2017), which can be considered as potential model-based biomarkers for healthy aging.

In summary, mathematical dynamical models can be built based on brain structure and activity derived from the empirical data and can potentially be applied to any topic addressed by data-driven analysis. The derived models can however provide additional information and even explanations of the observed phenomena, which can contribute to our understanding of brain dynamics and treatment of diseases.

## 3. Enhancing Data Analytics by Models

To build, validate and investigate models of brain networks, a number of computational tools have been developed including NEURON, NEST, BRIAN, and The Virtual Brain (TVB) (Carnevale and Hines, 2006; Gewaltig and Diesmann, 2007; Goodman and Brette, 2009; Sanz Leon et al., 2013), which combine biological relevance with up-to-date computational algorithms for high-performance computational clusters. For example, TVB is an open platform, where large-scale brain network models can be derived from the raw multi-modal imaging data, and an optimal match between empirical and simulated data, e.g., (dynamic) FC of RSNs, can be reached by an appropriate parameter calibration (Ritter et al., 2013; Sanz Leon et al., 2013; Sanz-Leon et al., 2015; Falcon et al., 2016).

The discussed dynamical models of brain networks are built on empirical SC establishing in such a way a natural link between brain structure and function. For sufficiently long scanning sessions, patterns of static FC of the spontaneous brain dynamics correlate with patterns of SC (Hagmann et al., 2008; Honey et al., 2009; Marrelec et al., 2016), and some properties of them can be inferred from each other (Hermundstad et al., 2013). This is also feasible for dynamic FC (Marrelec et al., 2016; Cabral et al., 2017b). However, the largely invariant SC can support many different FC patterns making the interdependence between them rather complicated (Park and Friston, 2013; Marrelec et al., 2016; Cabral et al., 2017a). For example, FC patterns most frequently observed in healthy older adults with good cognitive performance are less correlated with SC as compared to the connectivity patterns of poorly performing subjects (Cabral et al., 2017b).

Models can help to understand the correspondence between structural and functional connectivity (Honey et al., 2009). Homogeneous coupling strength and delay in coupling may lead to a strong structure-function correlation, whereas distributed delays and coupling strength between network nodes may cause a discrepancy between the observed activity patterns and underlying connectivity (Popovych et al., 2013; Ton et al., 2014). Therefore, one conclusion derived from theses modeling studies is that SC used for data analytics and mathematical modeling should be as precise as possible, especially, with regard to strength, delay and directionality of connections. This was also addressed by comparing large-scale brain simulations with models built on weighted undirected and directed structural connections (Knock et al., 2009), where the simulation in the latter case demonstrated more biologically realistic results.

Since investigation of the models is not restricted by the measured data, model parameters can be varied in a broad range to mimic possible scenarios of the evolution of brain states. For example, by modeling the hypothesis of structural disconnection in schizophrenia (Friston and Frith, 1995; Calhoun et al., 2009; Lynall et al., 2010; van den Heuvel and Fornito, 2014), parameters of the computational model can be varied from the states of strong to weak coupling or even by completely removing anatomical connections in the model (Cabral et al., 2012a). Such a computational experiment revealed changes in FC patterns also observed in empirical data (Lynall et al., 2010) and led to conclusion that disconnection-related neuropathologies can induce qualitatively similar changes in resting-state brain activity (Cabral et al., 2012a). From the opposite perspective, the modeling approach can be used to optimize SC configurations to obtain the best fit with FC from measured data by adding a few additional links and rewiring existing connections (Deco et al., 2014a). Such an approach may strongly contribute to the determination of empirical SC by predicting and suggesting possible anatomical links and their characteristics that are difficult to measure or to estimate from empirical data.

The above examples illustrate how the modeling can contribute to a mechanistic explanation of the structural and functional properties of the brain and their interrelation. With such an approach the emergent brain dynamics including resting state can be explained in terms of a few generic bifurcations (Cocchi et al., 2017) and assigned to a particular range of parameters that may vary depending on the brain state (Deco et al., 2013, 2017; Jobst et al., 2017). These parameters may have a well-founded relation to the brain characteristics such as global and local coupling, communication delay, noise intensity, heterogeneity of individual node dynamics and its excitation-inhibition balance as well as external input or stimulation (Ghosh et al., 2008; Deco et al., 2009, 2014b; Popovych and Tass, 2012). It is thus very important to perform a detailed investigation of the derived models, which could be assisted by the computational tools mentioned above. Several attempts in this direction have explored the emergence of multistability in the large-scale brain models (Deco and Jirsa, 2012; Golos et al., 2015; Hansen et al., 2015). The concepts of multi- and meta-stability, which distinguish themselves by the coexistence of either attractors or saddles, respectively (Cocchi et al., 2017), are the main model-based hypotheses of the emergence of the resting-state fluctuations of the BOLD signals and FC matrices (Deco et al., 2012, 2017; Deco and Kringelbach, 2016).

Furthermore, models can help to address the question on how exactly the brain parcellation (number and density of network nodes) and local connectivity utilized for the analysis of the brain dynamics may affect the results (Proix et al., 2016). This issue plays a crucial role for the data-driven studies, where several parcellation schemes (atlases) are currently in use without a general consensus on their appropriateness (Zalesky et al., 2010; Eickhoff et al., 2018a). On the other hand, the level of the parcellation granularity, for example, may strongly impact the topological properties of brain networks already at the level of SC (Zalesky et al., 2010). At the level of functional interrelations between brain nodes, the directionality, weight and type of connections are important, which can be addressed by an intrinsically model-based approach of dynamic causal modeling (DCM) (Friston et al., 2003) providing an estimation of effective connectivity reflecting causal interactions. Albeit inferring the effective connectivity in large-scale whole-brain networks is a challenging problem, recent progress with DCM (Friston et al., 2014, 2017; Frassle et al., 2017) may support a prompt development in this field.

Data analysis of the resting-state fMRI strongly relies on pre-processing steps, where confound regression is applied to clean fMRI recordings from contaminating signals (Murphy et al., 2013; Satterthwaite et al., 2013; Power et al., 2014; Salimi-Khorshidi et al., 2014; Varikuti et al., 2017). The validation of the brain models also depends on the respective nuisance signal regression, and previous modeling studies showed that conclusion derived from the models may depend on the utilized data processing steps, in particular, on whether the global brain signal was regressed or not (Cabral et al., 2012b, 2013; Yang et al., 2014; Anticevic et al., 2015). For different confound regression and data processing approaches, the impact of those on the parameters of the validated models can be evaluated. In such a way the effects of the data pre-processing procedures can be parametrized, classified and compared to each other as well as to the results of the data-driven approach (Varikuti et al., 2017).

Dynamical models can be applied to the entire range of the subject cohorts ranging from personalized models optimized for a single individual to the models designed to investigate effects of ensemble averaging, where a few tens of subjects were usually considered in the latter case (Cabral et al., 2012a; Bettinardi et al., 2017). The explanatory and predictive power of the large-scale models may be enhanced if large subject cohorts get involved in the modeling analysis as it is for the data-driven approach (Satterthwaite et al., 2013; Finn et al., 2015; Liem et al., 2017). On the other hand, personalized dynamical brain models can be an important goal for the model-based approach because of their application potential for personalized diagnosis, prevention and treatment of diseases as exemplified by the concept of the virtual epileptic patient (Jirsa et al., 2017; Proix et al., 2017). Other recent modeling studies suggest that model parameters may serve as potential biomarkers for disease diagnosis (Cabral et al., 2012b, 2013; van Hartevelt et al., 2014; Falcon et al., 2016). Such personalized models can also be used for the development and computational testing of personalized treatment of diseases.

In summary, there are several promising applications of the model-based approach, which could advance neuroimaging data analytics and translational medicine. To achieve this, an interdisciplinary research on data analysis should also include expertise from nonlinear dynamical systems and mathematics, focus on detailed investigation of the models and compare their results with the data.

## Author Contributions

OP, TM, FH, and SE contributed to the conception of the work. OP and TM drafted the manuscript. All the authors revised the manuscript and approved the final version of the manuscript.

### Conflict of Interest Statement

The authors declare that the research was conducted in the absence of any commercial or financial relationships that could be construed as a potential conflict of interest.
